# Putrescine Production by *Latilactobacillus curvatus* KP 3-4 Isolated from Fermented Foods

**DOI:** 10.3390/microorganisms10040697

**Published:** 2022-03-24

**Authors:** Rika Hirano, Aiko Kume, Chisato Nishiyama, Ryosuke Honda, Hideto Shirasawa, Yiwei Ling, Yuta Sugiyama, Misaki Nara, Hiromi Shimokawa, Hiroki Kawada, Takashi Koyanagi, Hisashi Ashida, Shujiro Okuda, Mitsuharu Matsumoto, Hiroki Takagi, Shin Kurihara

**Affiliations:** 1 Faculty of Bioresources and Environmental Sciences, Ishikawa Prefectural University, 1-308 Suematsu, Nonoichi 921-8836, Japan; rikahirano@outlook.com (R.H.); nishi80.1106@gmail.com (C.N.); sugiyama.yuta@gunma-u.ac.jp (Y.S.); naramisaki1028@gmail.com (M.N.); makida.hiroki@gmail.com (H.K.); koyataka@ishikawa-pu.ac.jp (T.K.); h-takagi@ishikawa-pu.ac.jp (H.T.); 2 Faculty of Biology-Oriented Science and Technology, Kindai University, 930 Nishimitani, Kinokawa 649-6493, Japan; 1818320052b@waka.kindai.ac.jp (R.H.); 2133710009u@waka.kindai.ac.jp (H.S.); 2144710004v@waka.kindai.ac.jp (H.S.); ashida@waka.kindai.ac.jp (H.A.); 3 Dairy Science and Technology Institute, Kyodo Milk Industry Co., Ltd., 20-1 Hirai, Hinode 190-0182, Japan; a-kume@meito.co.jp (A.K.); m-matumoto@meito.co.jp (M.M.); 4 Medical AI Center, Niigata University School of Medicine, 2−5274 Gakkocho-dori, Niigata 951-8514, Japan; seraphwyl@med.niigata-u.ac.jp (Y.L.); okd@med.niigata-u.ac.jp (S.O.)

**Keywords:** polyamine, probiotics, whole-genome sequencing, lactic acid bacteria

## Abstract

Polyamines are aliphatic hydrocarbons with terminal amino groups and are essential for biological activities. It has been reported that polyamines have health-promoting effects in animals, such as the extension of lifespan by polyamine intake. The identification of a high polyamine-producing bacterium from foods could lead to the development of a novel probiotic candidate. We aimed to identify high polyamine-producing bacteria from food, and isolated and collected bacteria from vegetables and fermented foods produced in Japan. We successfully acquired *Latilactobacillus curvatus* KP 3-4 isolated from Kabura-zushi as a putrescine producing lactic acid bacteria. Comparing the polyamine synthesis capability of *L. curvatus* KP 3-4 with that of typical probiotic lactic acid bacteria and *L. curvatus* strains available from the Japan Collection of Microorganisms, it was found that only *L. curvatus* KP 3-4 was capable of exporting high levels of putrescine into the culture supernatant. The enhancement of putrescine production by the addition of ornithine, and whole-genome analysis of *L. curvatus* KP 3-4, suggest that putrescine is synthesized via ornithine decarboxylase. The administration of *L. curvatus* KP 3-4 to germ-free mice increased the concentration of putrescine in the feces.

## 1. Introduction

Polyamines are aliphatic hydrocarbons with terminal amino groups. There are three principal polyamines: putrescine, spermidine, and spermine. Polyamines are essential for cell proliferation. Numerous health-promoting effects of polyamines on experimental animals have been reported, such as extending life span [1,2,3,4,5], enhancing memory [6], improving cognition [5], and improving heart function [7], although research has also been conducted to reduce polyamines, which are found in high concentrations in proliferating cancer cells, with the aim of inhibiting cancer promotion [8]. The biochemical mechanisms of health promotion by polyamines include the inhibition of oxidative stress [2], deacetylation of histone H3 through the inhibition of histone acetyltransferases [2], promotion of autophagy [2], and the suppression of abnormal gene methylation via an increase in the DNA methyltransferase activity [4]. Additionally, polyamines are important for the maintenance of human health through the regulation of the microbiota, as polyamines are used as cell-to-cell signals in *Proteus mirabilis*, a common urinary tract pathogen [9].

There are three sources of polyamines in animals: oral intake, production by intestinal bacteria, and endogenous biosynthesis. It has been reported that the amount of polyamines biosynthesized in the body decreases with aging [10]. In 2009, it was reported that when mice were fed chow containing differing polyamine concentrations, the concentration of polyamines in the blood was higher, the survival rate increased, and the incidence of glomerulosclerosis decreased when mice were fed chow with high polyamine content compared to when mice were fed chow with low polyamine content [1]. The amount of polyamines in the high-polyamine diet (365 mg/g) used in this previous study was about six times higher than that in the low-polyamine diet (66 mg/g).

In recent years, a study was conducted to investigate the effect of polyamine intake on health in elderly people [11]. In this previous epidemiologic study conducted by Wirth and colleagues, the polyamine group received 1.2 mg more polyamines per day from supplements containing polyamines derived from wheat germ, compared to the placebo group. It is reported that the estimated daily intake of dietary polyamines by humans is 42 mg/day in Europe (UK, Italy, Spain, Finland, Sweden, and The Netherlands) [12], 29 mg/day in the US [13], 16 mg/day in Turkey [14], 26 mg/day in Japan [15], and 36 mg/day in Sweden [16]. In the study by Wirth et al. [11], the change given by an additional 1.2 mg of polyamine derived from wheat germ, which is one of the foods that contain polyamines in the highest concentration [10], was only a few percent of the daily polyamine intake. Therefore, it would be necessary to explore and develop foods or supplements that contain polyamines with higher concentrations. The development of food containing high concentrations of polyamines would enable the use of new polyamine sources for clinical trials, which could lead to the expansion of a variety of studies. As a result, it is expected that knowledge on the health-promoting effects of polyamines will increase.

The concentration of polyamines in fermented foods such as cheese [17] and wine [18] is high, and fermented bacteria are considered to be related to the accumulation of polyamines in the foods. We previously analyzed the polyamine biosynthetic ability of 13 species of bifidobacteria and reported that none of them released large amounts of polyamines extracellularly [19].

On the other hand, polyamine synthesis by lactic acid bacteria has been reported in *Levilactobacillus brevis* [20], *Latilactobacillus curvatus* [20,21], *Lentilactobacillus hilgardii* [22], and *Lactiplantibacillus plantarum* [22], but their numbers are still limited and lactic acid bacteria that synthesize polyamines are not abundant.

The biogenic amines produced by lactic acid bacteria have been studied, focusing on histamine and tyramine, which can cause toxic effects in humans [23,24]. However, there are no previous studies that have systematically analyzed the polyamine production in lactic acid bacteria used as probiotics [25]. Therefore, we conducted research on putrescine, spermidine, and spermine, amines that have been reported to have health-promoting effects.

If the lactic acid bacteria consumed as food can synthesize polyamines, it would be possible to create a probiotic with potentially more health-promoting effects, and to produce supplements containing high concentrations of polyamines from the culture supernatant. Therefore, the purpose of this study was to screen lactic acid bacteria capable of synthesizing polyamines from fermented foods and vegetables that have traditionally been produced and consumed in Japan, and to compare their ability to produce polyamines with that of other commonly used probiotic lactic acid bacteria.

## 2. Materials and Methods

### 2.1. Mice

Male germ-free mice (Jcl:MCH(ICR)[GF]) were purchased from CLEA Japan Inc. (Tokyo, Japan). The animals were bred at Kyodo Milk Industry Co., Ltd. (Tokyo, Japan). The mice were housed in flexible film plastic isolators under a 12-h light/dark cycle at 25 ± 2 °C with 50% ± 10% humidity, with sterilized bedding, and were provided with sterilized water and sterilized low-polyamine pellet chow (modified AIN-93M formula with soybean oil in substitution of corn oil; Oriental Yeast Co., Ltd., Tokyo, Japan) ad libitum. The Kyodo Milk Animal Use Committee approved the protocols (permit number: 2020-23), performed in accordance with the Guide for the Care and Use of Laboratory Animals, published by the National Academies Press.

### 2.2. Bacterial Strains

With the exception of bacteria isolated from food, bacteria were obtained from the Japan Collection of Microorganisms (JCM) and from the American Type Culture Collection (ATCC). Bacteria were cultured at 37 °C in an anaerobic chamber (10% CO_2_, 10% H_2_, and 80% N_2_; InvivO_2_ 400, Ruskinn Technology, Bridgend, UK).

### 2.3. Gnotobiotic Mouse Experiment

Germ-free mice (*n* = 3) aged 4 weeks were orally administered sterilized phosphate-buffered saline (PBS) containing *Latilactobacillus curvatus* KP 3-4 (2.8 × 10^8^ CFU). Fecal samples were collected before oral administration on Day 0 and after 10 days of oral administration. *L. curvatus* KP 3-4 was streaked on a Gifu anaerobic medium (GAM) plate and anaerobically incubated at 37 °C for 48 h.

### 2.4. Isolation of Lactic Acid Bacteria from Food

Three methods (A to C, as described below) were used to isolate the lactic acid bacterial strains from the food. The methods used for each strain are shown in Table 1.

### 2.5. Screening for Polyamine-Producing Bacteria

Bacterial strains were inoculated on an MRS plate and were cultured anaerobically at 37 °C for 2 days to obtain the seed culture. Single colonies were inoculated into 500 µL of MRS broth and incubated anaerobically for 2 days at 37 °C. Five hundred μL of culture was centrifuged (18,700× *g*, 10 min, 4 °C) to separate the cells and culture supernatant. The concentration of polyamines in the supernatant was measured using high-performance liquid chromatography (HPLC).

### 2.6. Culture of Representative Probiotic Lactic Acid Bacteria and L. curvatus

Bacterial strains were inoculated to MRS broth in 96-deep-well plates from frozen glycerol stock and incubated at 37 °C for 38 h, and were used as pre-cultures. The pre-cultures were inoculated into 500 μL of fresh MRS broth in 96-deep-well plates using a copy plate stand (Tokken, Chiba, Japan). Growth was monitored by measuring the optical density at 600 nm (OD_600_). Four hundred μL of culture was centrifuged (18,700× *g*, 10 min, 4 °C) to separate the cells and culture supernatant. The collected cells were washed twice with 400 μL of PBS.

### 2.7. Culturing of L. curvatus KP 3-4 in Medium Supplemented with Ornithine

*L. curvatus* KP 3-4 was inoculated to MRS broth from frozen glycerol stock and was incubated at 37 °C for 24 h and used as pre-cultures. The pre-cultures were inoculated into 500 μL of fresh MRS broth supplemented with up to 1.2 mM ornithine in 96-deep-well plates using a copy plate stand (Tokken, Chiba, Japan). Growth was monitored by measuring OD_600_. The concentration of putrescine in the culture supernatant was then measured by HPLC.

### 2.8. Measurement of Polyamine Concentration in Culture Supernatant by HPLC

**Figure 1:**
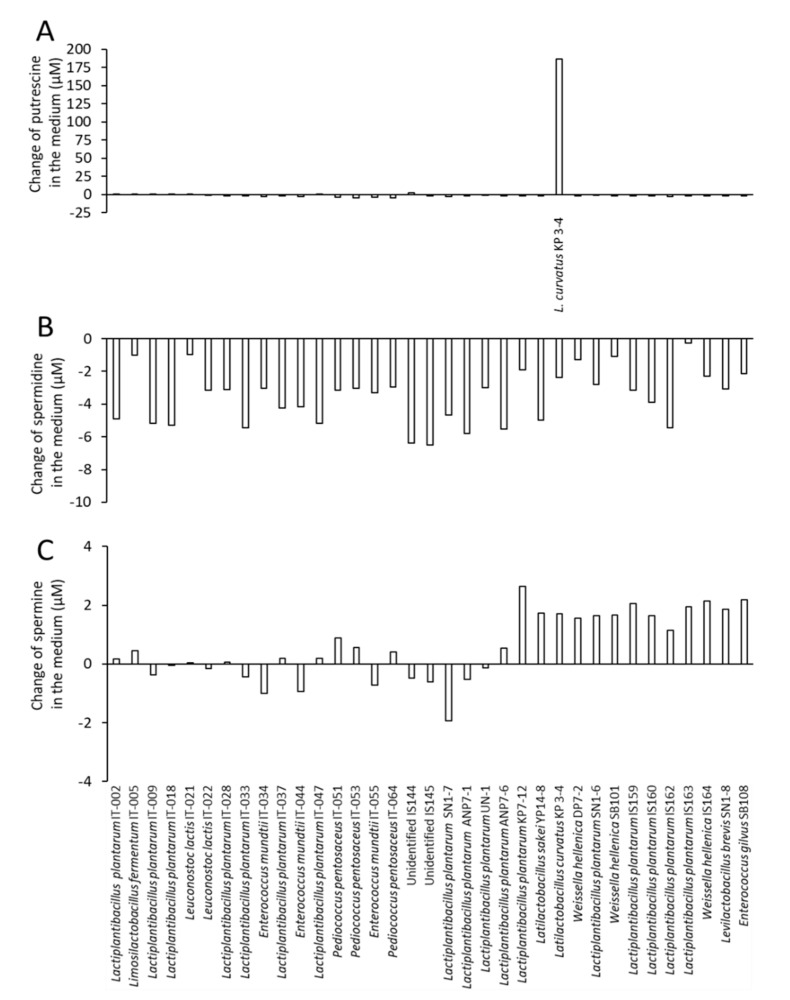
Concentration of polyamines in culture supernatants of bacteria isolated from food. Bacteria were isolated from food and cultured in MRS medium anaerobically for 2 days at 37 °C. Concentration of putrescine (**A**), spermidine (**B**), and spermine (**C**) in the culture supernatant was analyzed by HPLC (*n* = 1). The values are shown as changes in the amount of polyamine from the MRS medium to the culture supernatant.

**Figure 2:**
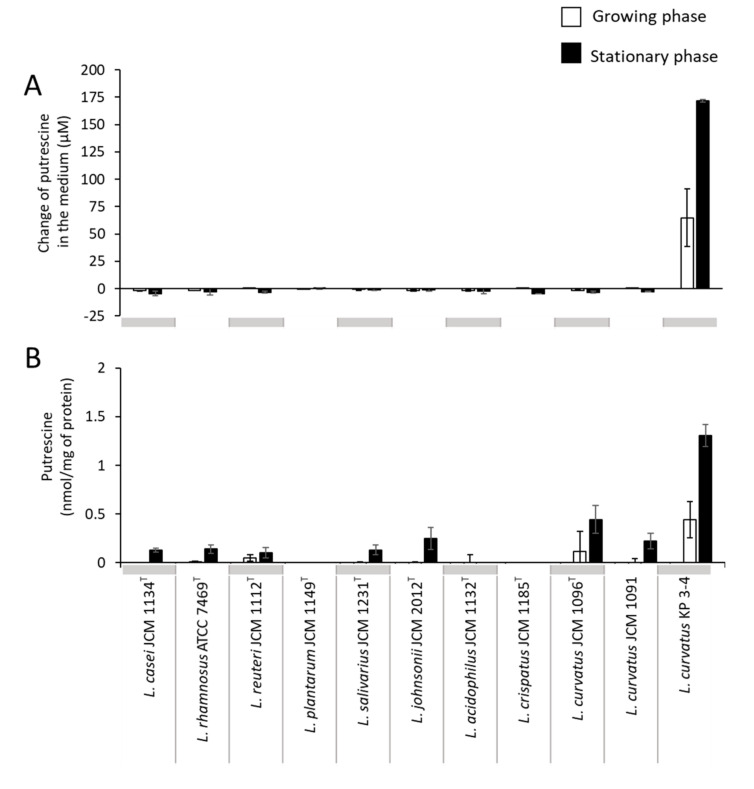
Putrescine concentration in culture supernatant and in the cells of lactic acid bacterial strains. Lactic acid bacterial strains were cultured in the MRS medium anaerobically for a maximum of 82 h at 37 °C. Putrescine concentration in culture supernatant (**A**) and intracellular putrescine concentration (**B**) of the tested lactic acid bacterial strains in the growing phase and stationary phase. For (**A**), the values are shown as changes in the amount of putrescine from the MRS medium to the culture supernatant. White bars, growing phase; black bars, stationary phase. Data represent means ± standard deviation from biological replicates (*n* = 3).

**Figure 3:**
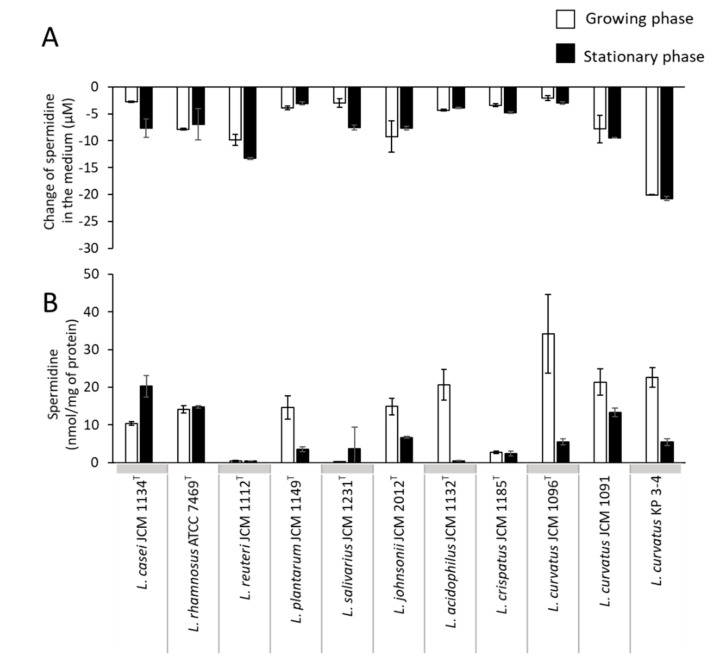
Spermidine concentration in culture supernatant and in the cells of lactic acid bacterial strains. Lactic acid bacterial strains were cultured in the MRS medium anaerobically for a maximum of 82 h at 37 °C. Spermidine concentration in culture supernatant (**A**) and intracellular spermidine concentration (**B**) of the tested lactic acid bacterial strains in the growing phase and stationary phase. For (**A**), the values are shown as changes in the amount of polyamine from the MRS medium to the culture supernatant. White bars, growing phase; black bars, stationary phase. Data represent means ± standard deviation from biological replicates (*n* = 3).

**Figure 4:**
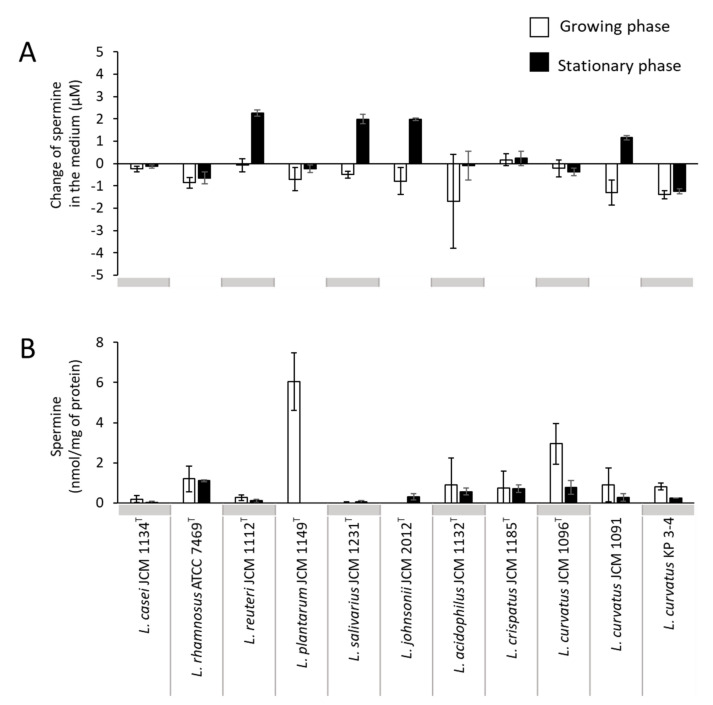
Spermine concentration in culture supernatant and in the cell of lactic acid bacterial strains. Lactic acid bacterial strains were cultured in the MRS medium anaerobically for a maximum of 82 h at 37 °C. Spermine concentration in the culture supernatant (**A**) and intracellular spermine concentration (**B**) of the tested lactic acid bacterial strains in the growing phase and stationary phase. For (**A**), the values are shown as changes in the amount of polyamine from the MRS medium to the culture supernatant. White bars, growing phase; black bars, stationary phase. Data represent means ± standard deviation from biological replicates (*n* = 3).

**Figure 5:**
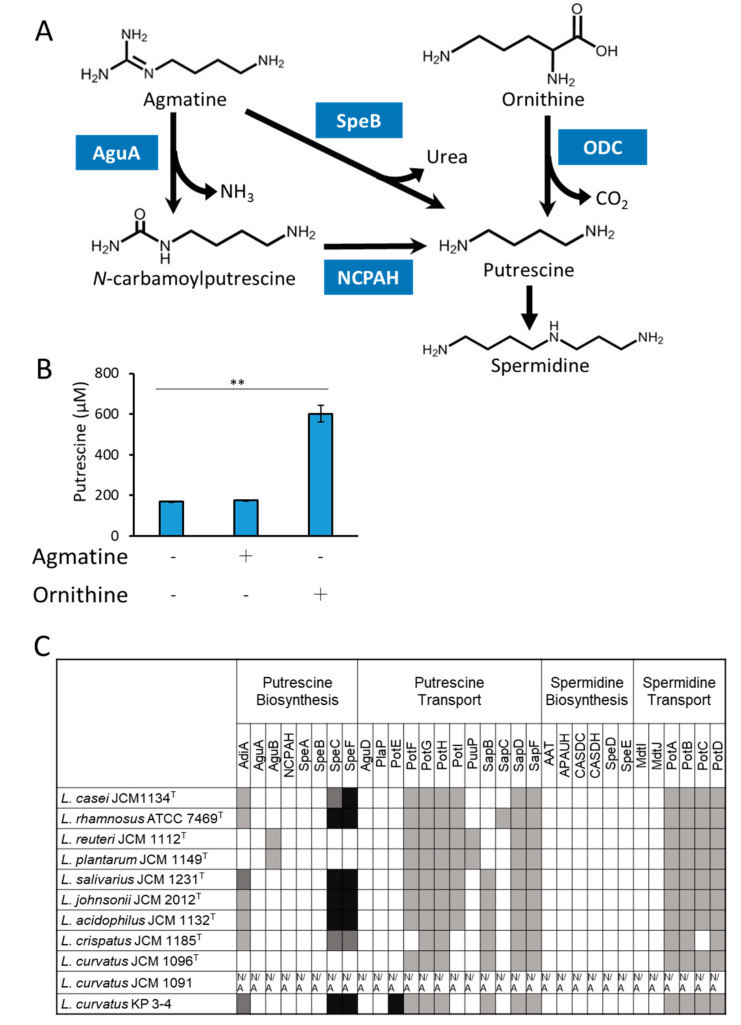
Prediction of the putrescine biosynthetic pathway by *L. curvatus* KP 3-4. (**A**) Known putrescine biosynthetic pathways in bacteria. The abbreviations used are as follows: AguA, agmatine deiminase; SpeB, agmatine ureohydrolase; NCPAH, *N*-carbamoylputrescine amidohydrolase; ODC, ornithine decarboxylase. (**B**) *L. curvatus* KP 3-4 was cultured in MRS or MRS with added arginine or ornithine anaerobically for 48 h at 37 °C and the amount of putrescine in the culture supernatant was analyzed by HPLC. Data represent means ± standard deviation from biological replicates (*n* = 3). Asterisks indicate statistical significance (** *p* < 0.01, Dunnett’s test with MRS as control). (**C**) Conservation analysis of polyamine synthesis and transport genes in lactic acid bacteria. The results shown are based on the tblastn analysis of the genome of the newly constructed *L. curvatus* KP 3-4 genome and genomes in the NCBI database (https://blast.ncbi.nlm.nih.gov/Blast.cgi (accessed on 16 May 2021)). Contig information for *L. curvatus* JCM1091 was not available in the NCBI database, and it is shown by N/A. AdiA, arginine decarboxylase; AguA, Agmatine deiminase; AguB, (AguB is NCPAH, putrescine carbamoyltransferase is PtcA); NCPAH, *N*-carbamoylputrescine amidohydrolase; SpeA, arginine decarboxylase; SpeB, agmatinase; SpeC, ornithine decarboxylase; SpeF, ornithine decarboxylase; AguD, agmatine:putrescine antiporter; PlaP, putrescine importer; PotE, putrescine:ornithine antiporter; PotF, putrescine ABC transporter periplasmic binding protein; PotG, ATP binding protein of putrescine ABC transporter PotFGHI; PotH, permease of putrescine ABC transporter PotFGHI; PotI, permease of putrescine ABC transporter PotFGHI; PuuP, putrescine importer; SapB, transmembrane protein of putrescine exporter SapBCDF; SapC, transmembrane protein of putrescine exporter SapBCDF; SapD, ATP binding protein of putrescine exporter SapBCDF; SapF, ATP binding protein of putrescine exporter SapBCDF; AAT, agmatine aminopropyltransferase; APAUH, aminopropylagmatine ureohydrolase; CASDC, carboxyspermidine decarboxylase; CASDH, carboxyspermidine dehydrogenase; SpeD, *S*-adenosylmethionine decarboxylase, proenzyme; SpeE, spermidine synthase; MdtI, multidrug efflux system transporter; MdtJ, multidrug efflux system transporter; PotA, ATP binding protein of spermidine/putrescine ABC transporter PotABCD; PotB, permease of spermidine/putrescine ABC transporter PotABCD; PotC, permease of spermidine/putrescine ABC transporter PotABCD; PotD, spermidine/putrescine ABC transporter. The color of each column indicates the score from the Protein BLAST analysis: (black) >500 bits, (dark gray) 500–300 bits, (light gray) 300–100 bits, and (white) <100 bits, respectively.

**Figure 6:**
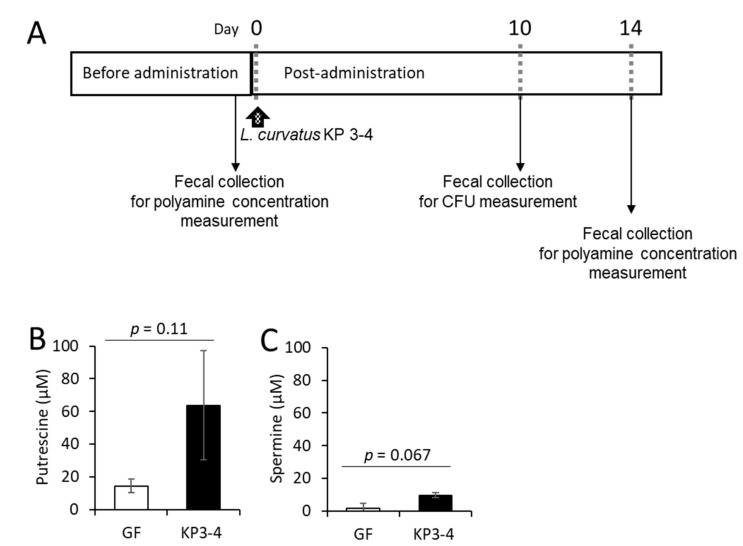
Effect of *L. curvatus* KP 3-4 colonization on polyamine levels in the intestines of mice. (**A**) The experimental schedule. Germ-free mice were administered *L. curvatus* KP 3-4 (*n* = 3). The arrows indicate the timing of administration. (**B**,**C**) Concentration of putrescine (**B**) and spermine (**C**) in the feces of mice before and after the administration of *L. curvatus* KP 3-4. Data represent means ± standard deviation. The *p* value was calculated by paired *t*-test.

After adding 1/10 volume of 100% (*w*/*v*) trichloroacetic acid to the collected culture supernatant, it was centrifuged (21,500× *g*, 10 min, 4 °C). The supernatants obtained from the previous centrifugation step were filtered using a Cosmonice filter W (0.45 μm, Nacalai Tesque, Kyoto, Japan) before the HPLC analysis. HPLC analysis was performed using previously described procedures [29]. For measuring the concentration of polyamines in the culture supernatant of the bacteria (Figures 1–5), the standard solution of each polyamine dissolved in MRS was used. In quantifying the polyamine concentration of the mouse feces (Figure 6) and culture supernatant of *L. plantarum* (Figure S2), polyamine dissolved in pure water was used as the standard.

### 2.9. Measurement of Concentration of Intracellular Polyamines by HPLC

The collected cells were resuspended in 500 μL of 5% (*w*/*v*) trichloroacetic acid and were incubated in boiling water for 20 min. After incubation on ice for 5 min and centrifugation (18,700× *g*, 20 min), the supernatants were filtered through the cosmo nice filter-W and subjected to HPLC analysis. Polyamine concentrations in the cells were normalized as previously reported [29]. Briefly, protein debris resulting from centrifugation after trichloroacetic acid and boiling treatment was dissolved in 0.1 N NaOH, and the protein concentration was measured by the Bradford method using the Bio-Rad Protein Assay (#5000006JA; Bio-Rad, Hercules, CA, USA). The resulting concentration of intracellular polyamines was determined as nmol/mg cellular protein.

### 2.10. Whole Genome Sequencing and De Novo Assembly of L. curvatus KP 3-4

Genomic DNA of *L. curvatus* KP 3-4 was extracted using Wizard^®^ Genomic DNA Purification Kit (A1620; Promega, Madison, WI, USA) following the manufacturer’s instructions. Here, 1 μg DNA was subjected to library construction using the 1D Genomic DNA Ligation Sequencing Kit (SQK-LSK 109, Oxford Nanopore Technologies, Oxford, UK) and sequenced on the MinION sequencer (Mk1B, Oxford Nanopore Technologies) according to the manufacturer’s protocol. The resulting ONT long reads were corrected with Canu ver 1.9 [30] using default configuration with the option of a total contig size of 3 Mb. The raw ONT reads were registered in DDBJ (accession number: DRA013072).

### 2.11. Conservation Analysis of Polyamine Biosynthetic Proteins and Transporters

Using the amino acid sequence described previously as a query sequence for a tblastn search [31], the sequence conservation of polyamine biosynthetic proteins and transporters was analyzed in the genomes of probiotic lactic acid bacteria described previously [25] and *L. curvatus*. The contig sequences used in the tblastn analysis are shown in Table 2. When the blast score was more than 500 bits, we determined that it was likely to be conserved.

### 2.12. Measurement of Polyamine Concentration in Mice Feces

The feces were diluted 10-fold by adding 9-fold weight of PBS to the feces and were suspended. Vortexing and incubation on ice were repeated three times. The suspension was centrifuged (18,700× *g*, 5 min) to obtain the supernatant. The concentration of polyamines in supernatant was measured using HPLC.

### 2.13. Statistical Analysis

Statistical analyses were performed using BellCurve for Excel (Social Survey Research Information Co., Ltd., Tokyo, Japan). In Figure 5B, statistical significance was determined using Dunnett’s test with MRS as the control. Three biological replicates were used for each group. In Figure 6, statistical significance was determined using paired *t*-test. Three mice were used. In all of the tests, *p* values of less than 0.05 were considered statistically significant: * *p* < 0.05, ** *p* < 0.01.

## 3. Results

### 3.1. Latilactobacillus curvatus KP 3-4 Derived from Kabura-Zushi Produces Putrescine

We first aimed to obtain polyamine-producing lactic acid bacteria from foods. After isolating 35 bacterial strains from vegetables and traditional fermented foods from Ishikawa and Wakayama prefectures in Japan (Table 1), the concentrations of putrescine, spermidine, and spermine in the culture supernatant cultured in the MRS medium were determined by HPLC. *Latiactobacillus curvatus* KP 3-4 from Kabura-zushi produced 187 µM putrescine in the culture supernatant and was screened as a candidate strain for putrescine production (Figure 1A), but high concentrations of putrescine were not detected in the culture supernatants of all of the isolated strains, except *L. curvatus* KP 3-4. In contrast, spermidine in the culture supernatant of all strains was reduced by several µM compared to that in the MRS medium (Figure 1B). Although spermine was increased in the culture supernatant of some strains, the changing amount was only a few µM (Figure 1C).

### 3.2. Comparison of Putrescine Productivity between L. curvatus KP 3-4 and Probiotic Lactic Acid Bacteria

To compare the polyamine synthesis and transport capability of *L. curvatus* KP 3-4 with other lactic acid bacteria and other *L. curvatus* strains (Table 3), we cultured well-known probiotic lactic acid bacteria [25] and three strains of *L. curvatus*, including the type strain (KP 3-4, JCM 1096^T^, and JCM 1091), in the MRS medium (Supplementary Figure S1) and analyzed the amount of polyamines in the culture supernatant using HPLC. The results showed that only *L. curvatus* KP 3-4, which was isolated in this study, produced putrescine at concentrations of up to 170 µM in the culture supernatant (Figure 2A). A small amount of putrescine was present in the cells of many strains, except for *L. plantarum*, *Lactobacillus acidophilus*, and *Lactobacillus crispatus*, with a maximum of 1.3 nmol/mg of protein. The amount of intracellular putrescine was the largest in *L. curvatus* KP 3-4 (Figure 2B).

Spermidine in the culture supernatant of all of the strains tested was reduced by several µM compared to that in the MRS medium (Figure 3A). In contrast, spermidine was present in the cells of most strains, except for *Limosilactobacillus reuteri* subsp. *reuteri* (Figure 3B). The amount was the highest in *L. curvatus* JCM1096^T^, with 34 nmol/mg of protein (Figure 3B).

Low concentrations of spermine were detected in the culture supernatant of four strains in the stationary phase (*L. reuteri* JCM 1112^T^, *Ligilactobacillus salivarius* JCM 1231^T^, *Lactobacillus johnsonii* JCM 2012^T^, and *L. curvatus* JCM 1091), although some strains showed reduced concentrations in the culture supernatants of the growing phase compared to the medium concentration of MRS (Figure 4A). In addition, a small amount of spermine (up to 6.0 nmol/mg of protein) was detected in the cells of several strains, and the amount tended to be higher during the growing phase (Figure 4B).

The MRS medium used for polyamine production evaluation contained about 8.3 ± 9.5 µM, 13.7 ± 1.0 µM, and 1.2 ± 1.1 µM of putrescine, spermidine, and spermine, respectively. For *L. plantarum*, the amounts of polyamines in the culture supernatant decreased as the culturing time passed (Supplementary Figure S2A–C). At the same time, there was no putrescine in the cells of *L. plantarum* (Supplementary Figure S2D), but there was spermidine and spermine in the cells (Supplementary Figure S2E,F).

### 3.3. Analyses of the Mechanism of Putrescine Production by L. curvatus KP 3-4

In bacteria, two putrescine biosynthetic pathways have been reported; one is from agmatine and the other is from ornithine (Figure 5A). In order to investigate the putrescine biosynthetic pathway in *L. curvatus* KP 3-4, this strain was cultured in MRS medium supplemented with agmatine or ornithine, and the concentration of putrescine in the culture supernatant was measured (Figure 5B). The putrescine production was over 3.5-times increased (from about 170 µM to about 600 µM) when cultured in the medium supplemented with ornithine compared to when cultured in the MRS medium (Figure 5B). The putrescine production by *L. curvatus* KP 3-4 increased up to a maximum of 94 mM with increasing the ornithine supplementation (Supplementary Figure S3). There was no change in putrescine production in the medium supplemented with agmatine (Figure 5B). These results suggested that *L. curvatus* KP 3-4 produces putrescine by ornithine decarboxylation with ornithine decarboxylase (ODC).

Next, we assessed whether *L. curvatus* KP 3-4 encodes conserved homologs of known bacterial polyamine synthesis and transport system genes, including ODC. First, whole genome sequencing of *L. curvatus* KP 3-4 was performed, and a contig of approximately 1.9 Mb of presumed genomic DNA and a contig of approximately 51 kb of presumed plasmid were obtained. The conservation of genes encoding known polyamine synthesis and transport systems in the genome of *L. curvatus* KP 3-4 and reference strains of probiotic lactic acid bacteria species (Table 2) were analyzed by tblastn (Figure 5C). ODC homologs were observed in seven out of the ten strains analyzed, including *L. curvatus* KP 3-4. The only strain with a high conservation of both ODC and putrescine exporter, PotE, was *L. curvatus* KP 3-4. Although the known biosynthetic genes for spermidine were not conserved in the genomes of any of the bacterial strains, most of the strains showed possible conservation of PotA, PotB, PotC, and PotD, the component proteins of the spermidine transporter PotABCD.

Comparative genomics using the genome of *L. curvatus* JCM 1096^T^ as the reference sequence (accession ID: NZ _CP026116) and that of *L. curvatus* KP 3-4 revealed that a 5586-bp region containing *odc* and *potE* was deleted in *L. curvatus* JCM 1096^T^ (Supplementary Figure S4A,B).

### 3.4. Oral Administration of L. curvatus KP 3-4 Increases Polyamine Levels in Feces of Mice

To investigate the potential of *L. curvatus* KP 3-4 as a probiotic capable of producing putrescine in the gut, we administered *L. curvatus* KP 3-4 to germ-free mice and compared the fecal polyamine levels before and after colonization (Figure 6A). After 10 days of administration of *L. curvatus* KP 3-4, the viable cell count in the feces was 8.7 × 10^8^ CFU/g feces, showing that the germ-free mice were colonized by *L. curvatus* KP 3-4. The concentration of putrescine increased from 14 to 64 µM (*p* = 0.11, paired *t*-test) after *L. curvatus* KP 3-4 colonization compared to before *L. curvatus* KP 3-4 colonization. The concentration of spermine increased from 1.7 µM to 9.6 µM (*p* = 0.067, paired *t*-test) after *L. curvatus* KP 3-4 colonization compared to before *L. curvatus* KP 3-4 colonization. No spermidine was detected either before or after colonization.

## 4. Discussion

In the present study, we successfully obtained *L. curvatus* KP 3-4 as a putrescine producing lactic acid bacteria from Kabura-zushi, a traditional Japanese fermented food. We also showed that the administration of *L. curvatus* KP 3-4 to germ-free mice increased the concentration of putrescine in the feces. Therefore, *L. curvatus* KP 3-4 may be a candidate for a new safe probiotic with health-promoting effects mediated by polyamines if safety and human studies can be conducted in the future to demonstrate efficacy.

In addition, based on the increase in putrescine production by the supplementation of ornithine (Figure 5B) and the high conservation of the ODC gene encoded by the genome (Figure 5C), we hypothesized that *L. curvatus* KP 3-4 produces putrescine by ODC. *L. curvatus* KP 3-4 also encodes a highly conserved PotE (Figure 5C), an ornithine/putrescine antiporter, suggesting that KP 3-4 may import ornithine via PotE, synthesize putrescine via ODC, and export putrescine via PotE.

The *odc* gene was conserved in the genome of *L. curvatus* KP 3-4 but not in the genome of the type strain, *L. curvatus* JCM 1096^T^ (Figure 5C and Supplementary Figure S4A,B). By focusing on the ability to produce putrescine, only KP 3-4 produced putrescine in the three strains cultured in this study among three *L. curvatus* strains: JCM 1096^T^, JCM 1091, and KP 3-4 (Figure 2A). In a previous report, when 15 strains of *L. curvatus* were compared, only three strains produced putrescine [20]. Therefore, putrescine production in *L. curvatus* is limited to certain strains, suggesting that ODC is conserved only in some strains and may have been acquired by horizontal gene transfer.

Focusing on food-derived *L. curvatus*, there was a report that *L. curvatus* derived from sausage produced putrescine [21]. On the other hand, there have been no studies on the probiotic effect of food-derived lactic acid bacteria on the host through the production of putrescine. We successfully isolated *L. curvatus* KP 3-4 from the fermented food Kabura-zushi, a traditional fermented food from Ishikawa Prefecture in Japan, which is made by sandwiching salted yellowtail between salted turnips and soaking them in malted rice for fermentation. We further showed that *L. curvatus* KP 3-4 produces polyamines in the intestine by colonization in mice. It has been reported that polyamines derived from gut microbes alleviated intestinal inflammation in mice [32], and gut microbes capable of producing polyamines are expected to improve the health of animals. If *L.* *curvatus* KP 3-4 can produce polyamines in the human intestine, and possibly improve health, *L*. *curvatus* KP 3-4 has the potential to become a breakthrough probiotic.

In addition, safety testing is required for *L. curvatus* KP 3-4 to be acceptable as a probiotic. There is no evidence that *L. curvatus* KP 3-4 does not produce histamine or tyramine, which can cause toxic effects in humans and animals. Therefore, these substances need to be analyzed in detail during safety testing.

In this study, spermidine was detected in the bacterial cells of almost all of the tested lactic acid bacteria, except for *L. reuteri* JCM 1112^T^ (Figure 3B), although none of the strains were predicted to have the known spermidine biosynthetic genes (Figure 5C). In addition, the amount of spermidine in the medium of all of the tested bacterial cultures was decreased (Figure 3A). When *L. plantarum* JCM 1149^T^ was cultured in MRS broth, the amounts of polyamines decreased in the medium, and polyamines were detected in the bacterial cells (Supplementary Figure S2). *potA*, *potB*, *potC*, and *potD*, which are known components of the bacterial spermidine transport system, were conserved in the genome of each lactic acid bacteria (Figure 5C). Therefore, the lactic acid bacterial strains in which spermidine was detected in the cells may have imported extracellular polyamines into the cells via the spermidine transporter PotABCD.

Previous reports of polyamines produced by lactic acid bacteria have been mostly limited to putrescine, but we analyzed the production ability of spermidine and spermine in addition to putrescine comprehensively for a well-known probiotic bacterial type strain. Our study provides the groundwork for the development of a potentially health-promoting probiotic, based on delivering bacterially-produced putrescine to the gastrointestinal tract.

## Figures and Tables

**Table 1:** List of strains for the screening of polyamine-producing bacteria.

Strains	Isolated Source	Isolation Method (A–C), GenBank Accession Number, or Reference
*Lactiplantibacillus plantarum* IT-002	cabbage	A
*Limosilactobacillus fermentum* IT-005	spinach	A
*Lactiplantibacillus plantarum* IT-009	mandarin orange	A
*Lactiplantibacillus plantarum* IT-018	cabbage	A
*Leuconostoc lactis* IT-021	spinach	A
*Leuconostoc lactis* IT-022	spinach	A
*Lactiplantibacillus plantarum* IT-028	mandarin orange	A
*Lactiplantibacillus plantarum* IT-033	lettuce	A
*Enterococcus mundtii* IT-034	lettuce	A
*Lactiplantibacillus plantarum* IT-037	onion	A
*Enterococcus mundtii* IT-044	lettuce	A
*Lactiplantibacillus plantarum* IT-047	onion	A
*Pediococcus pentosaceus* IT-051	mandarin orange	A
*Pediococcus pentosaceus* IT-053	broccoli	A
*Enterococcus mundtii* IT-055	broccoli	A
*Pediococcus pentosaceus* IT-064	mandarin orange	A
Unidentified IS144	Japanese Greens, Mibuna	A
Unidentified IS145	Japanese Greens, Mibuna	A
*Lactiplantibacillus plantarum* SN1-7	Nare-zushi using mackerel	B
*Lactiplantibacillus plantarum* ANP7-1	Nare-zushi using horse-mackerel	AB666313, [26]
*Lactiplantibacillus plantarum* UN-1	Nare-zushi using Japanese dace	B
*Lactiplantibacillus plantarum* ANP7-6	Nare-zushi using horse-mackerel	AB666315, [26,27]
*Lactiplantibacillus plantarum* KP7-12	Kabura-zushi	B
*Latilactobacillus sakei* YP14-8	Yamahai (yeast starter)	B
*Latilactobacillus curvatus* KP 3-4	Kabura-zushi	B
*Weissella hellenica* DP7-2	Radish sushi	B
*Lactiplantibacillus plantarum* SN1-6	Nare-zushi using mackerel	B
*Weissella hellenica* SB101	Squid pickled in malted rice	LC136890, [27]
*Lactiplantibacillus plantarum* IS159	Nare-zushi using mackerel	C
*Lactiplantibacillus plantarum* IS160	Nare-zushi using mackerel	C
*Lactiplantibacillus plantarum* IS162	Nare-zushi using mackerel	C
*Lactiplantibacillus plantarum* IS163	Nare-zushi using mackerel	C
*Weissella hellenica* IS164	Nare-zushi using mackerel	C
*Levilactobacillus brevis* SN1-8	Nare-zushi using mackerel	B
*Enterococcus gilvus* SB108	Nare-zushi using horse-mackerel	B

A: Appropriate amounts of materials (vegetables, fruits, and pickles) were soaked in de Man, Rogosa, and Sharpe (MRS) broth and were incubated for 1–2 days, then the aliquot of the broth was spread onto an MRS plate and further cultured at 37 °C under aerobic or anaerobic conditions. Isolated colonies were picked. B: Respective fermented food samples were suspended in a sterilized 0.85% (*w*/*v*) NaCl solution and spread onto MRS medium plates to grow lactic acid bacteria. The colonies obtained after 2–3 days of anaerobic incubation at 30 °C were streaked on MRS plates, and subsequent liquid cultures were stored as frozen glycerol stock. Species were identified based on the nucleotide sequence of the V1—V3 region of the 16S ribosomal RNA gene (16S rDNA). C: One gram of food was made up to 10 mL with sterile PBS to obtain 10 × (*v*/*w*) solution. The samples were completely suspended by vortexing and stirring, and were then diluted to 10^2^, 10^3^, 10^4^, and 10^5^-fold by stepwise dilution using sterile PBS. Dilutions were spread on MRS plates and incubated anaerobically at 37 °C for 5 days. The colonies obtained were streaked onto an MRS plate, and were anaerobically incubated at 37 °C for 5 days to obtain pure isolates. Single colonies were inoculated into MRS broth and were incubated anaerobically at 37 °C and stored as frozen glycerol stock. The identification of isolated lactic acid bacteria species was performed as described previously using the V1—V3 region of the 16S rDNA sequence [28].

**Table 2:** List of contigs used for the tblastn analysis.

Species	Reference
*Lacticaseibacillus casei* JCM1134^T^	GCF_000829055.1_ASM82905v1_genomic.fna
*Lacticaseibacillus rhamnosus* ATCC 7469^T^	GCF_007990855.1_ASM799085v1_genomic.fna
*Limosilactobacillus reuteri* subsp. *reuteri* JCM 1112^T^	GCF_000010005.1_ASM1000v1_genomic.fna
*Lactiplantibacillus plantarum* subsp. *plantarum* JCM 1149^T^	GCF_014131735.1_ASM1413173v1_genomic.fna
*Ligilactobacillus salivarius* JCM 1231^T^	GCF_001435955.1_ASM143595v1_genomic.fna
*Lactobacillus johnsonii* JCM 2012^T^	GCF_001433975.1_ASM143397v1_genomic.fna
*Lactobacillus acidophilus* JCM 1132^T^	GCF_001591845.1_ASM159184v1_genomic.fna
*Lactobacillus crispatus* JCM 1185^T^	GCA_001311685.1_ASM131168v1_genomic.fna
*Latilactobacillus curvatus* JCM1096^T^	GCF_004101845.1_ASM410184v1_genomic.fna
*Latilactobacillus curvatus* JCM1091	No assembly

**Table 3:** List of strains used for comparison of the polyamine synthesis ability with *L. curvatus* KP 3-4.

Strains	Source
*Lacticaseibacillus casei* JCM1134^T^	JCM
*Lacticaseibacillus rhamnosus* ATCC 7469^T^	ATCC
*Limosilactobacillus reuteri* subsp. *reuteri* JCM 1112^T^	JCM
*Lactiplantibacillus plantarum* subsp. *plantarum* JCM 1149^T^	JCM
*Lactobacillus acidophilus* JCM 1132^T^	JCM
*Lactobacillus crispatus* JCM 1185^T^	JCM
*Ligilactobacillus salivarius* JCM 1231^T^	JCM
*Lactobacillus johnsonii* JCM 2012^T^	JCM
*Latilactobacillus curvatus* JCM1096^T^	JCM
*Latilactobacillus curvatus* JCM1091	JCM

## Data Availability

All data needed to evaluate the conclusions in this work are present in the paper and/or the Supplementary Materials.

## Supplementary Materials

The following supporting information can be downloaded at: https://www.mdpi.com/article/10.3390/microorganisms10040697/s1, Figure S1: Growth curves of lactic acid bacterial strains; Figure S2: Polyamine concentration in culture supernatant and in the cell of *L. plantarum* JCM 1149^T^; Figure S3: Changes in the putrescine production of *L. curvatus* KP 3-4 by the supplementation of ornithine.; Figure S4: Comparative genomics of *L. curvatus* JCM 1096^T^ and KP 3-4.
