# Supplementary material for: Putrescine Production by Latilactobacillus curvatus KP 3-4 Isolated from Fermented Foods
Source: Microorganisms. 2022 Mar 24;10(4):697. doi: 10.3390/microorganisms10040697 (PMC9026525; doi:10.3390/microorganisms10040697)
Supplement: Supplementary file 1 [file microorganisms-10-00697-s001.zip › Supplementary Figure Caption.pdf]

Supplementary Figure S1 Growth curves of lactic acid bacterial strains

Growth curve of lactic acid bacteria in MRS under anaerobic condition. Data represent means ± standard deviation from biological replicates (*n* = 3). The white arrowheads indicate the sampling point used for the growing phase and the black arrowheads indicate the sampling point used for the stationary phase.

Supplementary Figure S2 Polyamine concentration in culture supernatant and in the cell of *L. plantarum* JCM 1149^T

The concentration of polyamines in the MRS medium and the concentration of polyamines in the culture supernatant and bacterial cells of *L. plantarum* JCM 1149^T after 10 h and 30 h of incubation in the MRS medium were measured. (A-C) Polyamine concentration in MRS medium and culture supernatant; A: putrescine, B: spermidine, C: spermine. (D-F) Concentration of polyamines in the bacterial cell; D: putrescine, E: spermidine, F: spermine. Data represent means ± standard deviation from biological replicates (*n* = 3).

Supplementary Figure S3 Changes in the putrescine production of *L. curvatus* KP 3-4 by the supplementation of ornithine.

L. curvatus KP 3-4 was cultured in MRS medium supplemented with ornithine at each concentration (0 mM, 250 mM, 500 mM, 750 mM, 1000 mM, 1200 mM) anaerobically for 48 hours at 37 °C. (A) Growth at each supplement concentration. (B) Putrescine concentration in culture supernatant. Data represent means ± standard deviation from biological replicates (*n* = 3).

Supplementary Figure S4 Comparative genomics of *L. curvatus* JCM 1096^T and KP 3-4

(A) Comparative genomics of the type strain (NZ_CP026116) and KP 3-4 strain of *L. curvatus*. Regions with high homology are connected by red lines. (B) Schematic diagram of the region that is present in the genome of KP 3-4 and missing in the genome of the type strain, which contains a sequence with high homology to ornithine decarboxylase (SpeC/SpeF) and a sequence with high homology to a putrescine-ornithine antiporter (PotE).
