# Supplementary material for: Putrescine Production by Latilactobacillus curvatus KP 3-4 Isolated from Fermented Foods
Source: Microorganisms. 2022 Mar 24;10(4):697. doi: 10.3390/microorganisms10040697 (PMC9026525; doi:10.3390/microorganisms10040697)
Supplement: Supplementary file 1 [file microorganisms-10-00697-s001.zip › Supplementary Figures.pptx]

## Slide 1
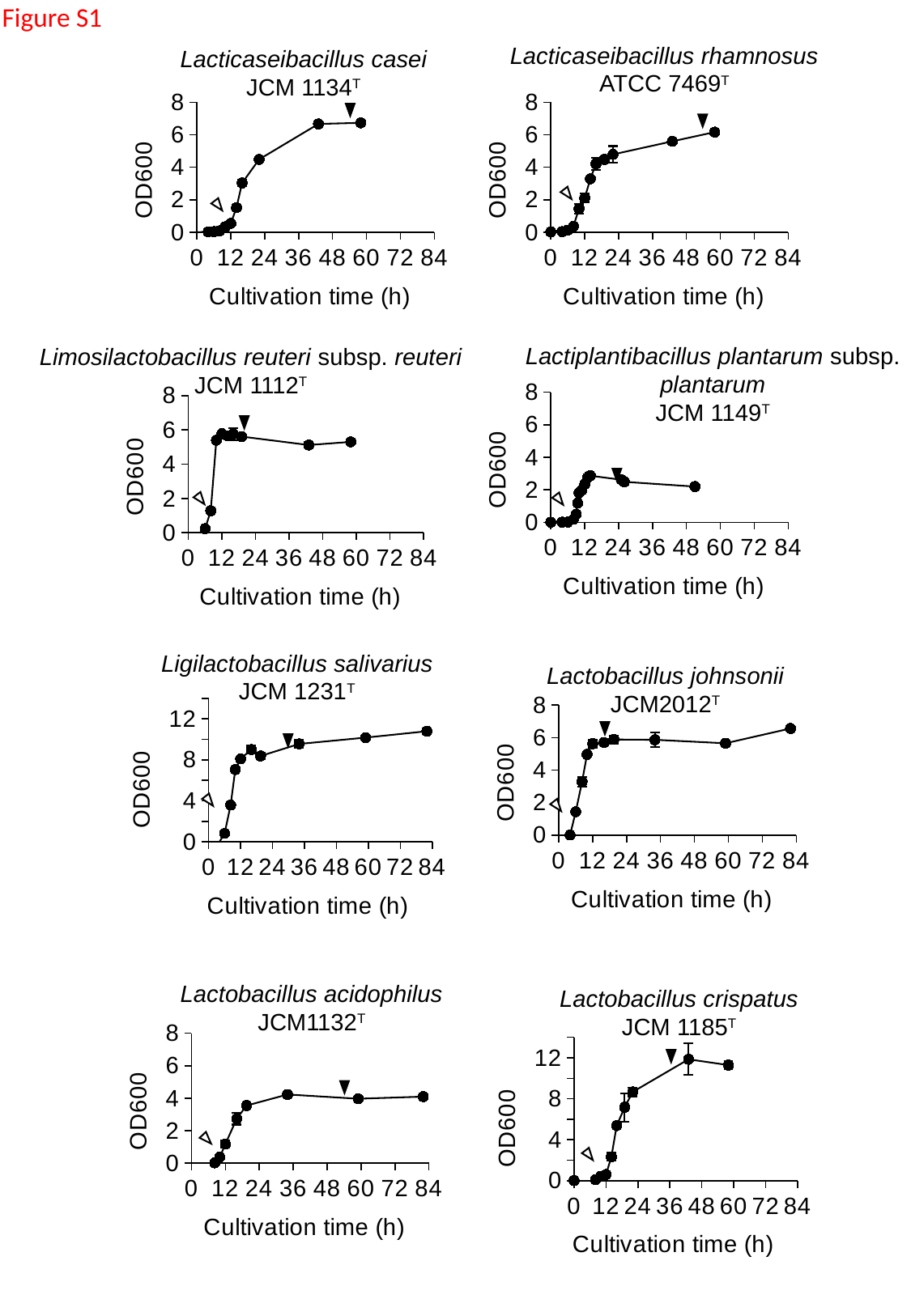

Figure S1
Lacticaseibacillus rhamnosus
ATCC 7469T
Lacticaseibacillus casei
JCM 1134T
### Chart
| Category | OD |
|---|---|
### Chart
| Category | OD |
|---|---|
Lactiplantibacillus plantarum subsp. plantarum
JCM 1149T
Limosilactobacillus reuteri subsp. reuteri
JCM 1112T
### Chart
| Category | OD |
|---|---|
### Chart
| Category | OD |
|---|---|
Ligilactobacillus salivarius
JCM 1231T
Lactobacillus johnsonii
JCM2012T
### Chart
| Category | OD |
|---|---|
### Chart
| Category | OD |
|---|---|
Lactobacillus acidophilus
JCM1132T
### Chart
| Category | OD |
|---|---|Lactobacillus crispatus
JCM 1185T
### Chart
| Category | OD |
|---|---|

## Slide 2
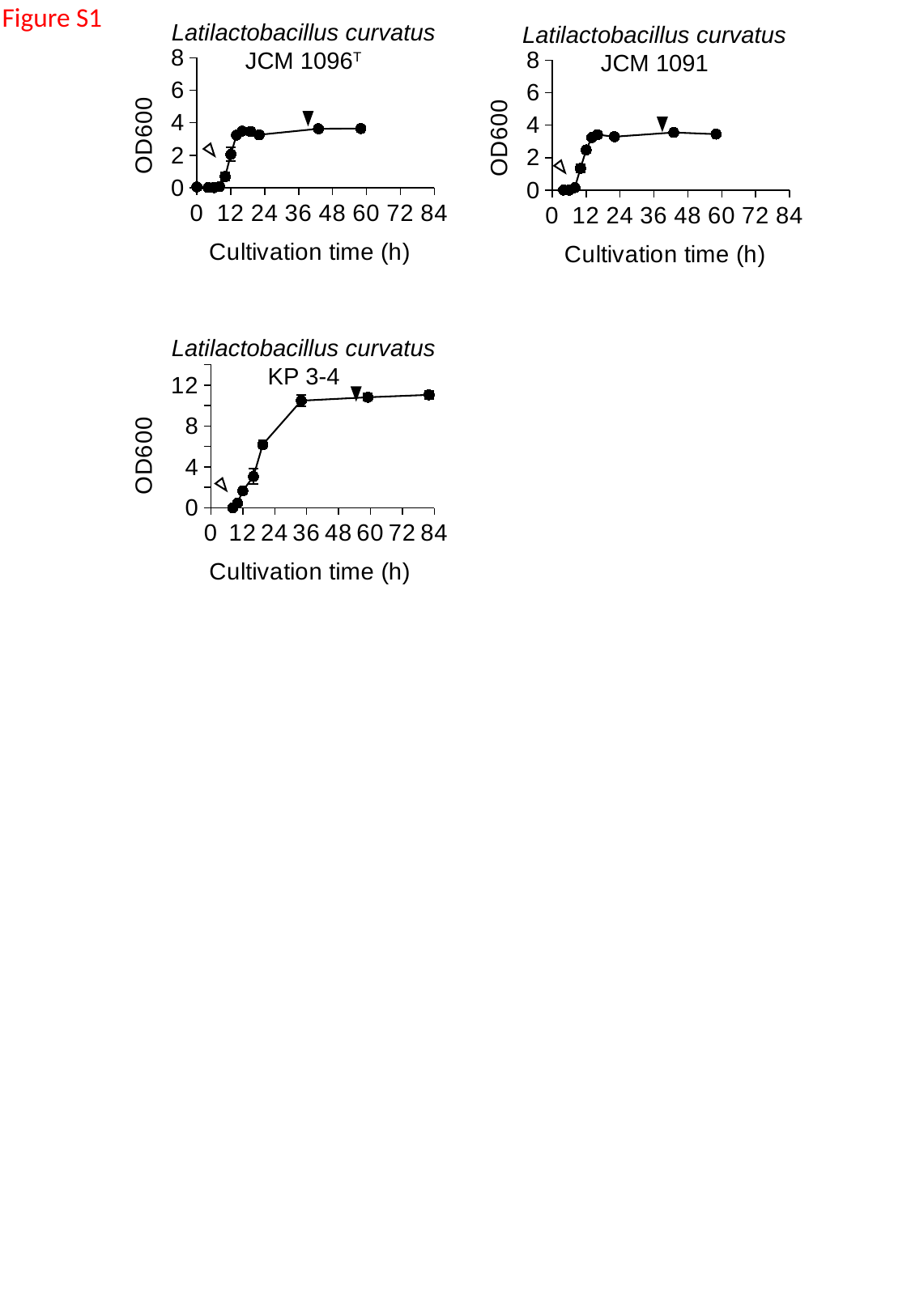

Figure S1
Latilactobacillus curvatus
JCM 1096T
Latilactobacillus curvatus
JCM 1091
### Chart
| Category | OD |
|---|---|
### Chart
| Category | OD |
|---|---|
Latilactobacillus curvatus
KP 3-4
### Chart
| Category | OD |
|---|---|

## Slide 3
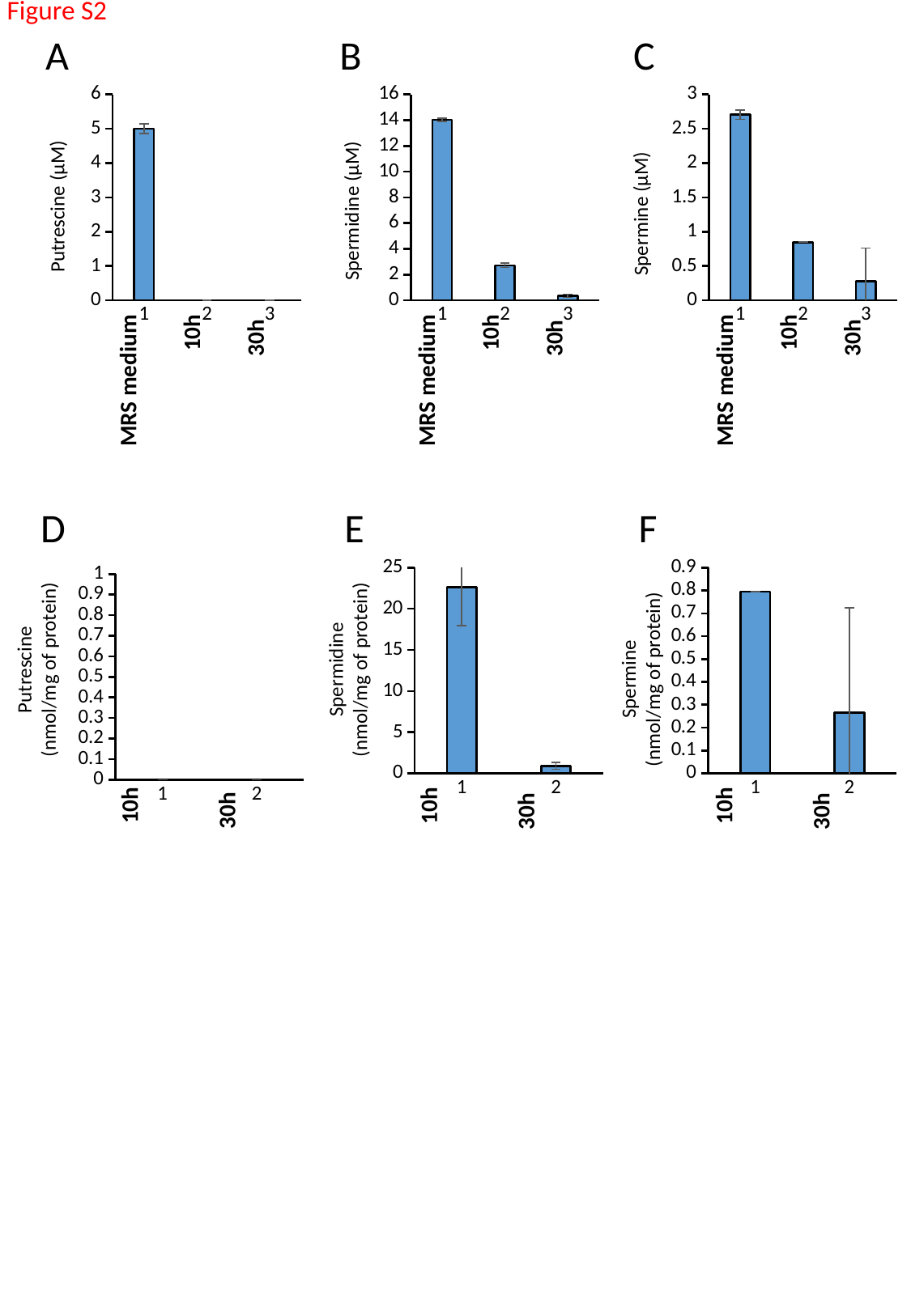

Figure S2
A
B
C
### Chart
| Category | 列1 |
|---|---|
### Chart
| Category | 列1 |
|---|---|
### Chart
| Category | 列1 |
|---|---|Putrescine (μM)
Spermine (μM)
Spermidine (μM)
| MRS medium | 10h | 30h |
| --- | --- | --- |
| MRS medium | 10h | 30h |
| --- | --- | --- |
| MRS medium | 10h | 30h |
| --- | --- | --- |
D
E
F
### Chart
| Category | 列1 |
|---|---|
### Chart
| Category | 列1 |
|---|---|
### Chart
| Category | 列1 |
|---|---|Putrescine
(nmol/mg of protein)
Spermidine
(nmol/mg of protein)
Spermine
(nmol/mg of protein)
| 10h | 30h |
| --- | --- |
| 10h | 30h |
| --- | --- |
| 10h | 30h |
| --- | --- |

## Slide 4
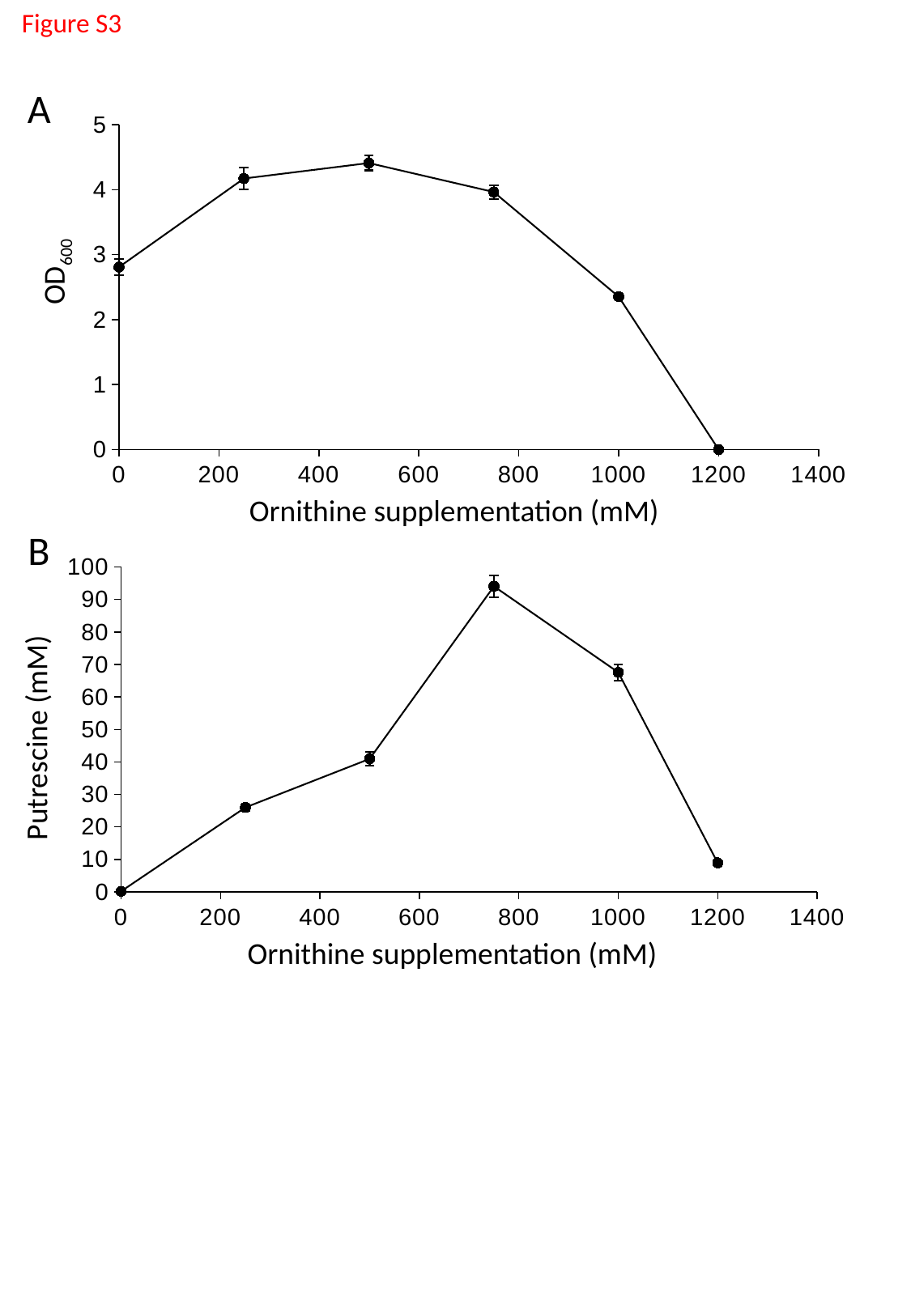

Figure S3
A
### Chart
| Category | OD |
|---|---|OD600
Ornithine supplementation (mM)
B
### Chart
| Category | put濃度(mM) |
|---|---|Putrescine (mM)
Ornithine supplementation (mM)

## Slide 5
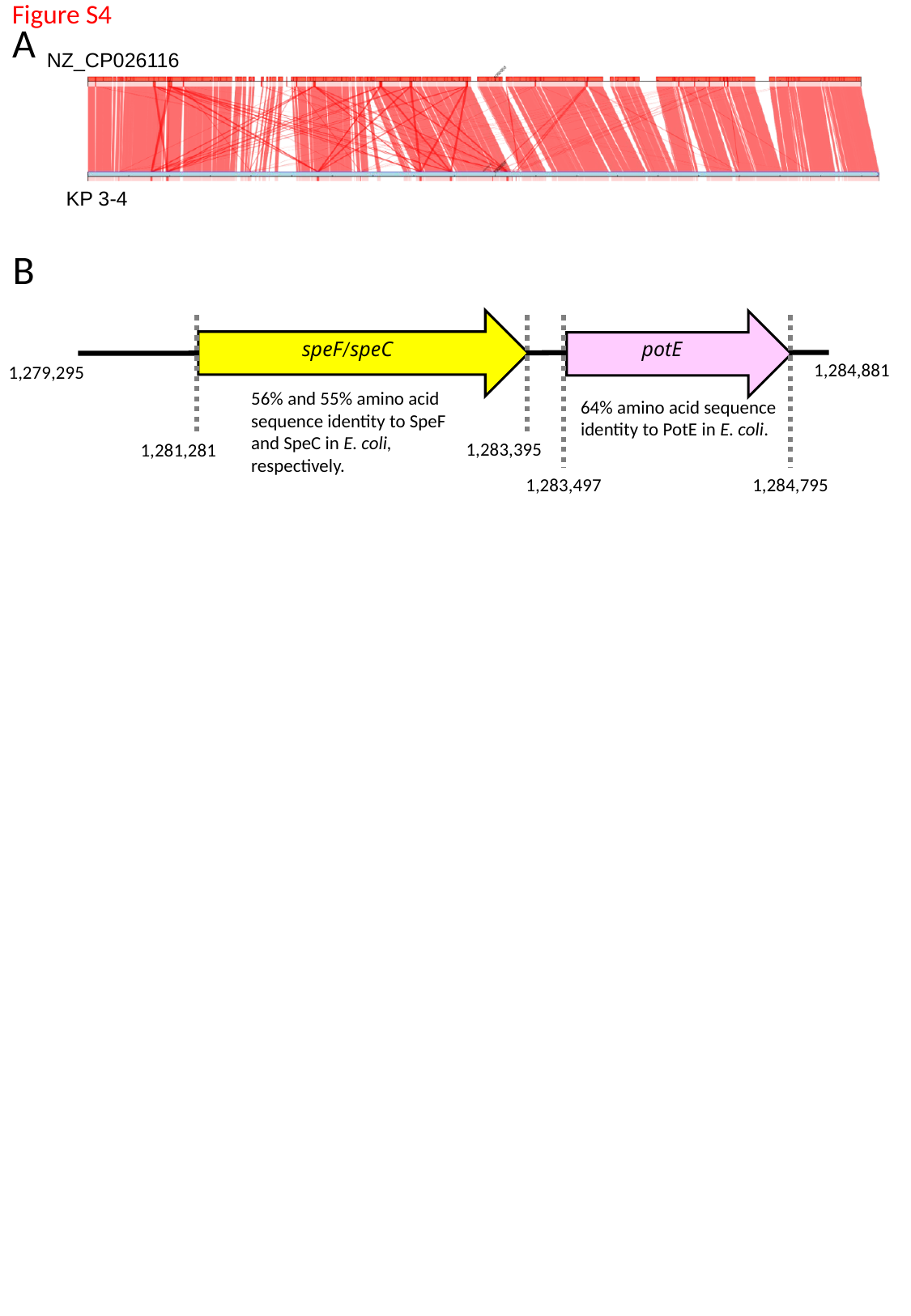

Figure S4
A
NZ_CP026116
KP 3-4
B
speF/speC
potE
1,284,881
1,279,295
56% and 55% amino acid sequence identity to SpeF and SpeC in E. coli, respectively.
64% amino acid sequence
identity to PotE in E. coli.
1,283,395
1,281,281
1,283,497
1,284,795
